# Cable properties and propagation velocity in a long single chain of simulated myocardial cells

**DOI:** 10.1186/1742-4682-4-36

**Published:** 2007-09-14

**Authors:** Lakshminarayanan Ramasamy, Nicholas Sperelakis

**Affiliations:** 1 Dept. of Electrical and Computer Engineering, University of Cincinnati College of Engineering, Cincinnati, OH, 45219, USA; 2 Dept. of Molecular & Cellular Physiology, University of Cincinnati College of Medicine Cincinnati, OH, 45267-0576, USA

## Abstract

**Background:**

Propagation of simulated action potentials (APs) was previously studied in short single chains and in two-dimensional sheets of myocardial cells [1-3]. The present study was undertaken to examine propagation in a long single chain of cells of various lengths, and with varying numbers of gap-junction (g-j) channels, and to compare propagation velocity with the cable properties such as the length constant (*λ*).

**Methods and Results:**

Simulations were carried out using the PSpice program as previously described. When the electric field (EF) mechanism was dominant (0, 1, and 10 gj-channels), the longer the chain length, the faster the overall velocity (*θ*_ov_). There seems to be no simple explanation for this phenomenon. In contrast, when the local-circuit current mechanism was dominant (100 gj-channels or more), *θ*_ov _was slightly slowed with lengthening of the chain. Increasing the number of gj-channels produced an increase in *θ*_ov _and caused the firing order to become more uniform. The end-effect was more pronounced at longer chain lengths and at greater number of gj-channels.

When there were no or only few gj-channels (namely, 0, 10, or 30), the voltage change (ΔV_m_) in the two contiguous cells (#50 & #52) to the cell injected with current (#51) was nearly zero, i.e., there was a sharp discontinuity in voltage between the adjacent cells. When there were many gj-channels (e.g., 300, 1000, 3000), there was an exponential decay of voltage on either side of the injected cell, with the length constant (*λ*) increasing at higher numbers of gj-channels. The effect of increasing the number of gj-channels on increasing *λ *was relatively small compared to the larger effect on *θ*_ov_. *θ*_ov _became very non-physiological at 300 gj-channels or higher.

**Conclusion:**

Thus, when there were only 0, 1, or 10 gj-channels, *θ*_ov _increased with increase in chain length, whereas at 100 gj-channels or higher, *θ*_ov _did not increase with chain length. When there were only 0, 10, or 30 gj-channels, there was a very sharp decrease in ΔV_m _in the two contiguous cells on either side of the injected cell, whereas at 300, 1000, or 3000 gj-channels, the voltage decay was exponential along the length of the chain. The effect of increasing the number of gj-channels on spread of current was relatively small compared to the large effect on *θ*_ov_.

## Background

Successful transmission of excitation from one myocardial cell to the next contiguous cell can occur without the necessity of gj-channels between the cells. This has been demonstrated to be possible in theoretical and modeling studies by Sperelakis and colleagues [1-5], and has been confirmed by other laboratories [6-8]. As was stated in the 1977 paper of Sperelakis and Mann [4], for the EF mechanism to work successfully, the junctional membrane must be more excitable than the contiguous surface sarcolemma. The fact that the junctional membranes (i.e., the intercalated disks) have a higher concentration (density) of fast Na^+ ^channels than the surface sarcolemma should cause them to be more excitable than the surface membrane. In a simulation study of cardiac muscle, Kucera et al. [9] determined how conduction velocity varied with the fraction of fast I_Na _channels located in the junctional membranes. For a 10 nm (100 Å) cleft width and 50 % of the I_Na _channels located in the junctional membranes, they found that conduction still occurred at a velocity of about 20 cm/sec when cell coupling was reduced to 10 % of normal and at about 10 cm/sec when coupling was only 1 % of normal. In biological studies on connexon43 knockout mice, absent in gj-channels in their hearts, propagation velocity was only slowed and not blocked [10-12]. Although the presence of gj-channels is not essential for propagation of excitation in the heart, when hearts do contain gj-channels, propagation velocity is speeded up. The PSpice simulation studies suggest that too many gj-channels (e.g., more than 100 channels per junction) causes the propagation velocity to exceed the physiological range.

In previous studies on simulated myocardial cells, propagation of action potentials (APs) was examined in short chains of cells (e.g., 10 cells long) and in 2-dimensional sheets (e.g., 10 × 10 and 20 × 10), with the number of gj-channels varied from zero to 10,000 [1,4,13,14]. Propagation of excitation occurred at near-physiological speeds even when there were no gj-channels connecting between the longitudinally-oriented cells [1]. The mechanism proposed was the relatively large electric field (EF) that develops in the narrow junctional clefts when the prejunctional membrane fires an AP [1,4,13-15]. This EF action is accentuated when the junctional membranes contain fast Na^+ ^channels at a higher density than that in the surface sarcolemma [9,15,16]. Transverse propagation also occurred by the same EF mechanism between adjacent parallel chains that were closely packed [2,3].

The present study was undertaken to examine propagation in long single chains, in which the cells were connected by varying numbers of gj-channels, and to compare the propagation velocity with the measured cable properties, such as the length constant (*λ*). It was found that the effect of increasing the number of gj-channels on *λ *was relatively small compared to the large effect on propagation velocity. In addition, the present studies were undertaken to provide confirmation of the parameter values used in the model, as for example, the values of the input resistance and the length constant.

## Methods

Compared to other models, such as the mathematical model, a simulation study of cardiac muscle using PSpice provides the ability to change the electrical equivalence of physiological parameters. A simulation study by PSpice can be made as accurate as using the mathematical model. Additionally, PSpice provides the ability to vary the parameters at a discrete point in a chain of cardiac cells, whereas in the mathematical model this requires an extensive reconstruction of the circuit. Another important advantage of PSpice is its portability. That is, the model can be easily transferred from person to person for confirmation and for further studies. For example, we have submitted our model for publication in a website [17] for easy use by other investigators. In addition, we have provided similar information to numerous individuals who have contacted us. Furthermore, several other types of electrophysiological studies have been done recently using PSpice.

**Figure 1:**
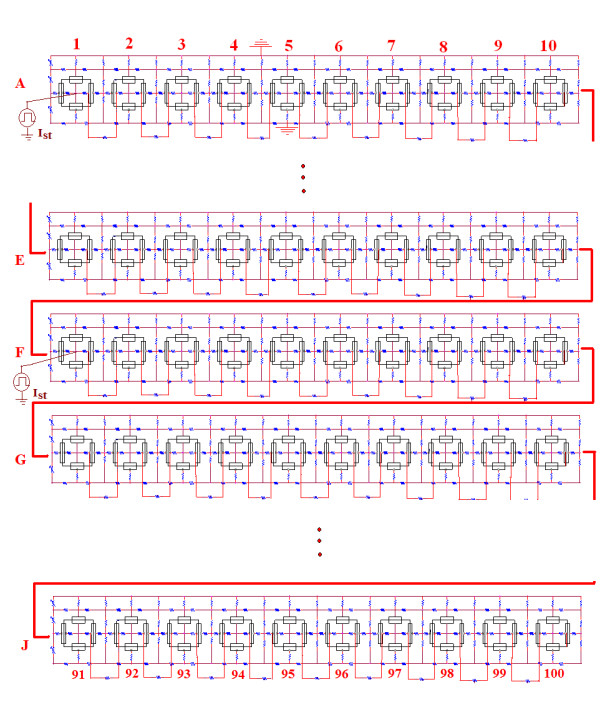
Schematic diagram for the single chain of 100 myocardial cells. Rows 2 (B), 3 (C), 4 (D), 8(H), and 9 (I) have been omitted in order to contain the size of the figure. For the propagation velocity experiment, cell #1 was stimulated intracellularly with rectangular depolarizing current pulses of 0.1 nA amplitude and 0.25 ms duration. The resultant action potentials (APs) were recorded only from cells 1, 10, 20, 30, 40, 50, 60, 70, 80, 90, and 100 in order to limit the number of traces. For the length constant experiments, intracellular depolarizing rectangular current pulses (10 nA, 5 ms) were applied to cell #51 (middle of chain), and the resulting voltage changes in cell #51 and its immediate neighbors were measured. For this type of experiment, the cells were made inexcitable by removing their GTABLEs.

The methods used for the PSpice simulations were thoroughly described in previous publications [1-3,18,19]. The essential difference is that the present study used long single chains of 10, 20, 50, and 100 cells. The 100-cell chain is depicted in Figure 1. All parameters used were those used previously, and they are summarized in Table 1. One variable was the number of gj-channels that was inserted between the contiguous cells (0, 1, 10, 30, 50, 70, 100, 300, 1000, or 3000). Each gj-channel was assumed to have a conductance of 100 pS. For the study on propagation velocity, Cell #1 was stimulated with intracellular rectangular depolarizing current pulses (0.1 nA, 0.25 ms).

**Table 1:** Parameter values used under standard conditions

**Parameters**	**Values**
C_m_	300 fF (30)
R_K_	71 MΩ (710)
R_Na_	710 MΩ (7100)
E_K_	-94 mV
E_Na_	+60 mV
R_d_	5000 MΩ
C_d_	30 pF
R_or_	1.0 KΩ
R_ol_	1.0 KΩ
R_i_	500 KΩ
R_jc_	25 MΩ

Values for junctional units given in parentheses

C_m _= Total cell capacitance

R_K _= Potassium resistance

R_Na _= Sodium resistance

E_K _= Potassium equilibrium potential

E_Na _= Sodium equilibrium potential

R_d _= Resistance in delay circuit for second black-box to bring about AP repolarization

C_d _= Capacitance in delay circuit for second black-box

R_or _= Radial resistance of external fluid

R_ol _= Longitudinal resistance of external fluid

R_i _= Longitudinal resistance of intracellular fluid

R_jc _= Radial resistance of junctional cleft

For study of cable properties, the basic units were rendered inexcitable by removal of their GTABLEs. The GTABLE is PSpice nomenclature for specifying how the conductance (or current) varies with membrane potential during excitation [1]. Intracellular current pulses (I_o_) were applied to the middle of the 100-cell chain (namely cell #51), and the fall-off of voltage was measured on both sides of the injected cell. The resulting potentials were plotted on linear and logarithmic ordinates (i.e., semilog plot) to measure the *λ *values. The *λ *values can also be calculated from the following equation:

ΔVx=ΔV0e−xλ
 MathType@MTEF@5@5@+=feaafiart1ev1aaatCvAUfKttLearuWrP9MDH5MBPbIqV92AaeXatLxBI9gBaebbnrfifHhDYfgasaacH8akY=wiFfYdH8Gipec8Eeeu0xXdbba9frFj0=OqFfea0dXdd9vqai=hGuQ8kuc9pgc9s8qqaq=dirpe0xb9q8qiLsFr0=vr0=vr0dc8meaabaqaciaacaGaaeqabaqabeGadaaakeaacqqHuoarcqWGwbGvdaWgaaWcbaGaemiEaGhabeaakiabg2da9iabfs5aejabdAfawnaaBaaaleaacqaIWaamaeqaaOGaemyzau2aaWbaaSqabeaadaWcaaqaaiabgkHiTiabdIha4bqaaGGaciab=T7aSbaaaaaaaa@3B6C@

where ΔV_x _is the voltage change at any distance x, ΔV_0 _is the voltage change at the point of current injection (x = 0), and *λ *is the length constant (in cm). Equation ^#^1 may be solved for *λ *by the following expression:

λ=x2.303log⁡Vo−2.303log⁡Vx
 MathType@MTEF@5@5@+=feaafiart1ev1aaatCvAUfKttLearuWrP9MDH5MBPbIqV92AaeXatLxBI9gBaebbnrfifHhDYfgasaacH8akY=wiFfYdH8Gipec8Eeeu0xXdbba9frFj0=OqFfea0dXdd9vqai=hGuQ8kuc9pgc9s8qqaq=dirpe0xb9q8qiLsFr0=vr0=vr0dc8meaabaqaciaacaGaaeqabaqabeGadaaakeaaiiGacqWF7oaBcqGH9aqpdaWcaaqaaiabdIha4bqaaiabikdaYiabc6caUiabiodaZiabicdaWiabiodaZiGbcYgaSjabc+gaVjabcEgaNjabdAfawnaaBaaaleaacqWGVbWBaeqaaOGaeyOeI0IaeGOmaiJaeiOla4IaeG4mamJaeGimaaJaeG4mamJagiiBaWMaei4Ba8Maei4zaCMaemOvay1aaSbaaSqaaiabdIha4bqabaaaaaaa@4923@

where 2.303 is the factor for converting natural logs to logs to the base of 10.

The input resistance (R_in_) was measured from the slope of the linear ΔV_m_/I_o _curve in the injected cell (#51). The polarization resistance (R_p_) was measured from the linear ΔV_m_/I_o _slopes in the contiguous cells. These V/I curves were linear, on both the depolarizing and hyperpolarizing sides, because rectification was not incorporated into the basic units.

The myocardial cells were assumed to be cylindrical in shape, 150 μm in length and 16 μm in diameter. The cleft width of the cell junctions (intercalated disks) was assumed to be 100 Å, and the radial shunt resistance of the junction to be 25 MΩ (50 MΩ/2) (Table 1).

## Results and Discussion

### A. Propagation velocity in single chains

#### 1. Variation in number of gj-channels (100-cell chain)

**Figure 2:**
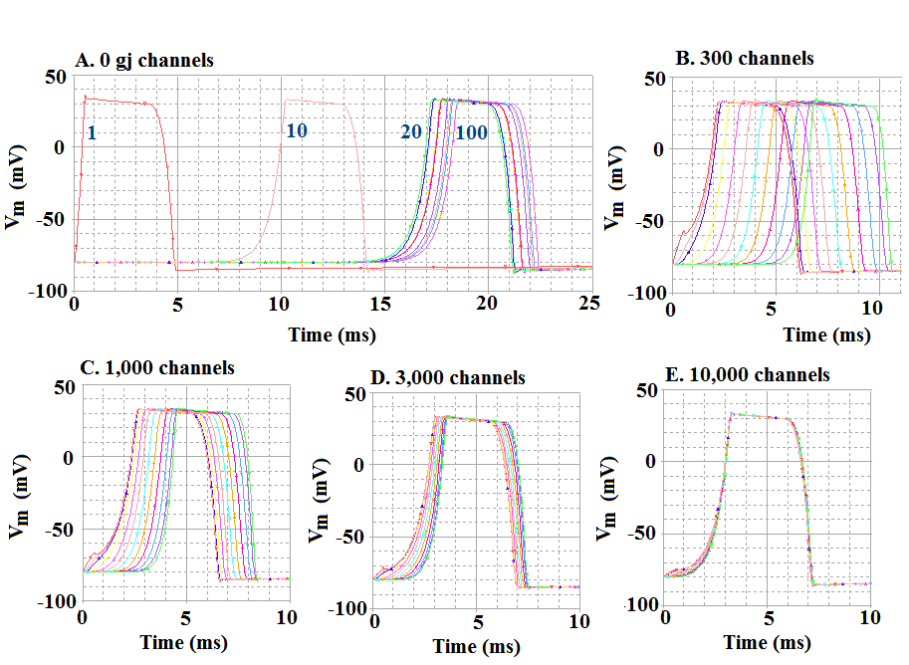
Propagation of simulated action potentials (APs) in a single linear chain of 100 myocardial cells. Cell #1 was stimulated intracellularly, and the resultant APs were recorded from only cells #1, 10, 20, 30, 40, 50, 60, 70, 80, 90, and 100 (to limit the number of traces). The number of gap-junction (g-j) channels at the cell junctions was varied over a wide range, but only five are illustrated, namely 0 gj-channels (A), 300 (B), 1,000 (C), 3,000 (D), and 10,000. The traces numbered in panel A are for APs recorded from cells #1, 10, 20, and 100; the remaining traces are bunched up between cells 20 and 100, some of them being nearly superimposed. Note that adding gj-channels markedly speeds up the velocity of propagation. In panel E, all 11 traces are superimposed.

**Figure 3:**
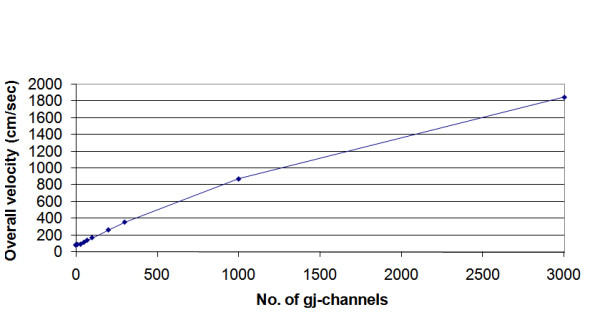
Graphic summary that quantitates how the propagation velocity in simulated cardiac APs varies with the number of gj-channels in the single chain of 100 cells. Note that relationship is nearly linear up to 300 gj-channels. Increasing the number of gj-channels 10-fold (from 100 to 1,000) increased velocity about 5-fold (5.2 fold).

The overall velocity of propagation (*θ*_ov_) increased markedly with an increase in number of gj-channels inserted at the cell junctions. These results are depicted in Figure 2. The number of gj-channels was varied over a very wide range, from 0 (complete EF mechanism for transmission of excitation from cell to cell) to 10,000 (0, 10, 30, 50, 70, 100, 200, 300, 1000, 3000, and 10,000). The records illustrated in Figure 2 are only for 0, 30, 1000, 3000 and 10,000 gj-channels. The propagation velocity became non-physiologically fast when there were 100 or more gj-channels (Fig. 3). "Non-physiologically fast" means that propagation velocity was considerably above values measured in the heart. As can be seen in Figure 3, there was a nearly linear relationship between *θ*_ov _and the number of gj-channels up to 300.

The propagation velocity at zero gj-channels varied from 10.6 cm/sec for a 10-cell chain, to 18.5 cm/sec for a 20-cell chain, to 46.0 cm/sec for a 50-cell chain, and to 95.5 cm/sec for a 100-cell chain (Table 2).

**Table 2:** Summary of effect of the number of gj-channels on overall propagation velocity (*θ*_ov_) in single chains of cells

**A. 100 cells**			
**No. of gj-channels**	***θ***_**ov**_	**Firing order**	**End-effect**

	**(cm/s)**	**(Rating)**	**(cells affected)**

0	*95.5	C	10 – 100
1	^#^92.0	C	20 – 100
10	102	B	50 – 100
30	117	A-	90 – 100
50	120	A	90 – 100
70	139	A	90 – 100
100	169	A	90 – 100
1000	833	A	90 – 100

**B. 50 cells**			

0	46.0	B	30 – 50
1	46.3	A-	Bunching
10	51.4	A	Yes (slight)
100	188	A	Yes

**D. 20 cells**			

0	18.5	C	No
1	23.1	A-	Bunching
10	53.6	A	Yes (slight)

**E. 10 cells**			

0	10.6	A-	9 – 10
1	19.2	A	9 – 10 (sl)

* *θ*_ov _was 91.5 cm/s when R_jc _was lowered to 5 MΩ (from std. of 50 MΩ)

# *θ*_ov _was slightly slowed by inserting one gj-channel

#### 2. Variation in length of single chain

**Figure 4:**
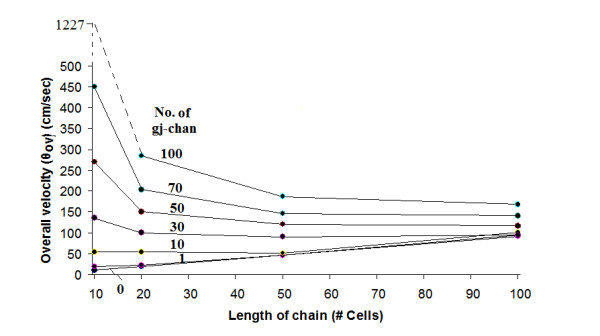
Graphic summary that quantitates how the overall propagation velocity varies with the length of the single chain. For this experiment, the 100-cell chain was shortened to 50, 20, and 10 cells, so that the results could be reliably compared, and the number of gj-channels in each chain length was varied from 0 to 100. The results show that when there was no or only few gj-channels (namely, 0, 1 or 10), propagation velocity increased with length of the chain. In contrast, when there were many gj-channels (e.g., 30, 50, 70, or 100), the velocity was slowed when chain length was increased, the most prominent effect occurring between chain lengths of 10 and 20 cells. As can be seen, the family of curves tended to converge at the chain length of 100 cells.

The length of the single chain was also varied, and different numbers of gj-channels were inserted. These results are depicted in Figure 4. The length of the chain was varied between 10 and 100 cells. As shown in Figure 4, when the EF mechanism was dominant (0, 1, or 10 gj-channels), propagation velocity increased almost linearly with increase in chain length. We believe that this phenomenon may be caused by an end-effect. When the local-circuit current mechanism was dominant (100 gj-channels or higher), then propagation velocity did not increase with chain length, and, in fact, there was a decrease (Fig. 4).

Our explanation for why *θ*_ov _decreases at longer chain lengths, when there are many gj-channels, is as follows. When there are 100 gj-channels, for example, and the chain length is short (e.g., 10 cells long), then the spread of the stimulation current extends over a greater fractional length of the chain, so those cells are simultaneously brought to threshold. Therefore, regular propagation need occur over only a few cells, so *θ*_ov _is larger. As the chain length is extended, this effect becomes smaller and smaller, so *θ*_ov _decreases.

Table 2 summarizes the propagation velocity data given in this section and section #1 above, and, in addition, summarizes the uniformity of firing and the end-effect that was observed. The end-effect is a phenomenon that is well known in electrical engineering and occurs at the end (or edge) of a long circuit containing repeat units and in cables that have a sharp termination point. We previously showed that end-effects do occur in our PSpice model [20]. As shown in Table 2, propagation velocity at 0, 1, or 10 gj-channels (EF mechanism dominant) was markedly slowed at shorter chain lengths, with values of about 10.6, 18.5, and 46.0 cm/sec for 0 gj-channels. These slower values are more in line with the values of 30–50 cm/sec previously reported for short chains and small 2-dimensional sheets when propagation was by the EF mechanism alone [1-3]. As reported previously, *θ*_ov _(for 0 gj-channels) was critically dependent on the value of R_jc_, the radial shunt resistance of the juntional clefts. In the present study, R_jc _was held fixed at 25 MΩ (50 MΩ/2).

As shown in Table 2, the firing order in a 100-cell chain became uniform when the number of gj-channels was increased to 30 or more. That is, when the EF mechanism was dominant (0, 1, or 10 gj-channels), the firing order was erratic. We believe that erratic firing was caused by a prominent end-effect. However, at shorter chain lengths, propagation became more uniform at no or few gj-channels. For example, at a chain length of 10 cells (Table 2, D), propagation was quite uniform even at 0 or 1 gj-channel.

There was some parallelism between uniformity of firing and the end-effect. The more disordered the firing, the greater the end-effect. For example, in Table 2A for the 100-cell chain, when the firing order was rated as very good ("A" rating), then the end-effect was confined to the last 10% of the chain (cells 90–100). In contrast, when the firing order was rated as poor ("C" rating), the end-effect was evident over the last 80–90% of the chain. When the chain length was shorter (B-D of Table 2), the end-effect, in general, was less pronounced.

### B. Voltage fall-off with distance in a 100-cell chain

#### 1. Length constant (*λ*) measurements

**Figure 5:**
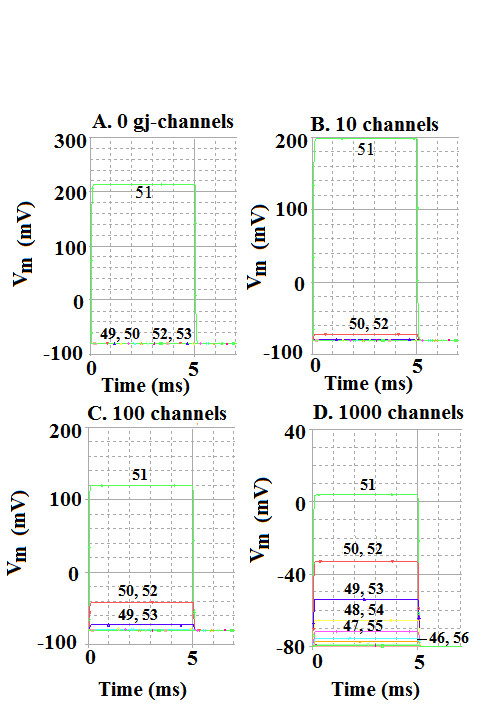
Experiment to measure the spread of current in the linear chain of 100 cells. The myocardial cells were rendered inexcitable by removing their GTABLEs. Depolarizing current pulses (10 nA, 5 ms) were applied intracellularly to cell #51 near the mid-point of the chain, and the resulting membrane voltage changes were recorded from cell #51 and its immediate neighbors (e.g., cells 44–58). The number of gj-channels was varied over a wide range (namely 0, 10, 30, 100, 300, 1000, and 3000), but the results from only 4 are illustrated in this figure: 0 gj-channels (A), 10 (B), 100 (C), and 1,000 (D). **A: **With no gj-channels, the voltage change in cell #51 was very large (ca 215 mV), whereas there was almost zero voltage change in the contiguous cells (49, 50, 52, 53). **B: **with 10 gj-channels, the ΔV_m _in cell 51 was ca 200 mV, whereas that in cells on either side (cells 50 & 52) was only about 8 mV. **C: **With 100 gj-channels, the ΔV_m _in cell 51 was ca 120 mV, that in cells 50 and 52 was ca 40 mV, and that in cells 49 and 53 was ca 8 mV. **D: **With 1000 gj-channels, the ΔV_m _in cell 51 was ca 84 mV and those in the contiguous cells were ca 44 mV (cells 50 & 52), ca 26 mV (cells 49 & 53), ca 15 mV (cells 48 & 54), and ca 8 mV (cells 47 & 55).

**Figure 6:**
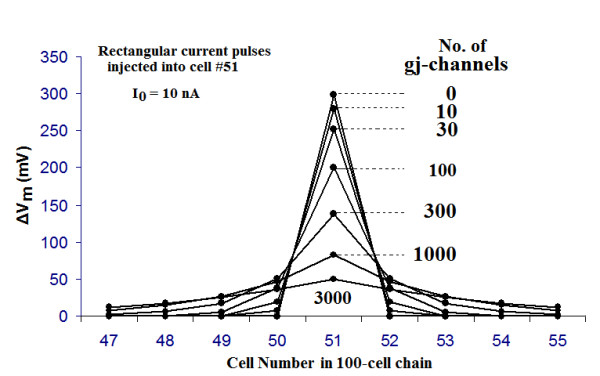
Graphic summary of the data collected from the experiments on the spread of current with various numbers of gj-channels (0, 10, 30, 100, 300, 1,000, and 3,000). Rectangular current pulses (10 nA, 5 ms) were injected intracellularly into cell #51 (near the middle of the linear chain of 100 cells), and the resulting membrane potential changes (ΔV_m_) were measured in the injected cell and its immediate neighbors. The myocardial cells were made inexcitable by removal of their GTABLEs. As can be seen, when there were no gj-channels, the ΔV_m _in the two contiguous cells (50 & 52) was nearly zero. When there were 10 or 30 channels, there was a small ΔV_m _in cells 50 and 52. When there were 300, 1000, or 3000 channels, the fall-off of ΔV_m _was exponential, i.e., the cells behaved like a long cable.

The decay in voltage as a function of distance along a single-chain of 100 cells was measured with various numbers of gj-channels inserted at the cell junctions (Fig. 5). The current (rectangular pulses) was injected intracellularly near the mid-point of the chain, namely into cell #51, and the transmembrane potential change (ΔV_m_) measured in the adjoining cells on both sides (Tables 3, 4). The original data traces are illustrated in Figure 5 for only four of the gj-channel numbers: 0, 10, 100, and 1000. Note that for 0 and 10 channels, there was a sharp discontinuity in ΔV_m _between the injected cell and its immediate neighbors. These results are plotted on a linear ordinate scale in Figure 6, and the actual values are listed in Tables 3, 4. When there were no or only a few gj-channels (0, 10, 30), the ΔV_m _in the contiguous cells on both sides was very small, being almost zero at 0 gj-channels. That is, there was a very sharp discontinuity in ΔV_m _between the injected cell and its immediate neighbors (Fig. 6). These results are consistent with what has been reported physiologically [21,22]. In such a situation, the length constant (*λ*) cannot be measured, being imaginary and less than the length of one cell (<150 μm).

**Table 3:** Summary of effect of number of gj-channels on the decay of voltage as a function of distance in a chain of 100 cells

	No. of gj-channels	I_o _(nA)	ΔV_o _(mV)	ΔV_x _(mV) Cell number			
				50 & 52	49 & 53	48 & 54	47 & 55

**A**	0	10.0	293	0	0	0	0
		5.0	148	0	0	0	0
		1.0	29.5	0	0	0	0
		0.5	14.8	0	0	0	0
**B**	1	10.0	295	0	0	0	0
		5.0	147	0	0	0	0
		1.0	29.5	0	0	0	0
		0.5	14.6	0	0	0	0
**C**	10	10.0	280	8.0	0	0	0
		5.0	140	4.0	0	0	0
		1.0	28.0	0.5	0	0	0
		0.5	14.0	0.2	0	0	0
**D**	100	10.0	200	40.0	3.0	0	0
		5.0	100	20.0	3.0	0	0
		1.0	20.0	4.0	0.5	0	0
		0.5	10.0	2.0	0.3	0	0
**E**	300	10.0	139	50.1	18.1	6.5	2.4
		5.0	69.3	25.0	9.0	3.3	1.2
		1.0	13.9	5.0	1.8	0.7	0.2
		0.5	6.9	2.5	0.9	0.3	0.1
**F**	1000	10.0	83.0	48.0	26.0	15.0	8.0
		5.0	42.0	23.0	13.0	7.0	4.0
		1.0	8.3	4.7	2.7	1.5	0.8
		0.5	4.2	2.3	1.3	0.7	0.4
**G**	3000	10.0	51.5	36.2	25.4	17.9	12.6
		5.0	25.7	18.1	12.7	9.0	6.3
		1.0	5.1	3.6	2.5	1.8	1.3
		0.5	2.6	1.8	1.3	0.9	0.6
**H**	10000	10.0	31.2	26.7	25.0	21.5	17.2
		5.0	15.6	13.3	12.5	10.8	10.0
		1.0	3.2	2.7	2.5	2.2	2.0
		0.5	1.6	1.3	1.3	1.1	1.0

Current was injected into cell #51 (middle of chain)

**Table 4:** Measurement of the spread of current in a 100-cell chain containing various numbers of gj-channels

	**ΔV**_m_**(mV)**						
	
**Cell Number**	**Number of gj-channels**						
	
	**0**	**10**	**30**	**100**	**300**	**1000**	**3000**
47	0	0	0	0	2.3	8.1	12.8
48	0	0	0	0	6.6	14.5	17.6
49	0	0	0	7.4	17.8	26.1	25.9
50	0	8	19.4	38.3	50.4	46.8	36.9
51	298	280	252	200	138 (50.9)	83.0 (30.5)	50.0 (18.4)
52	0	8	19.4	38.3	50.4	46.8	36.9
53	0	0	0	7.4	17.8	26.1	25.9
54	0	0	0	0	6.6	14.5	17.6
55	0	0	0	0	2.3	8.1	12.8

I_o _(10 nA) was injected in the middle of the chain, namely cell # 51.

V_x _= V_o _e^-*x*/*λ*^. When x = *λ*, V_x _= V_o _1/e = 0.368 V_o_.

Values given in parentheses (cell #51) are the 1/e values (0.368 x V_o_).

**Figure 7:**
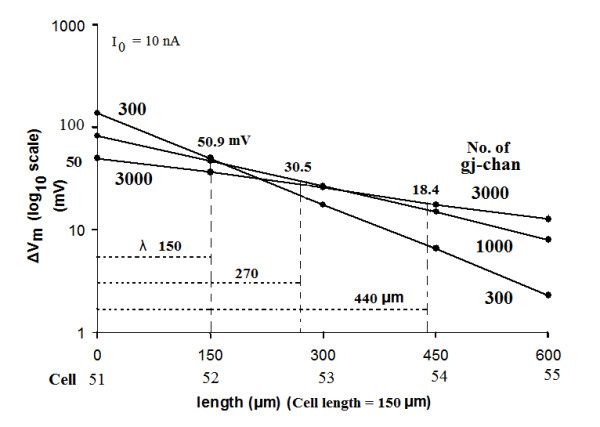
The length constant data obtained for 300, 1000, and 3000 gj-channels are plotted on a semi-logarithmic plot to illustrate that the data points form a straight line. The ordinate gives the ΔV_m _on a log scale, and the abscissa gives the distance along the cable (one direction only) from the point of current injection (middle of cell 51) and assuming the length of each myocardial cell to be 150 μm. Thus, the second labels on the abscissa give the cell number. The value of the length constant (*λ*) is the distance at which the voltage falls to 1/e (1/2.717) or 36.8 %. Thus, the following *λ *values were obtained: 150 μm (for 300 gj-channels), 270 μm (for 1000 gj-channels), and 440 μm (for 3000 gj-channels). Hence, increasing the number of gj-channels 10-fold (300 to 3000) increased *λ *about 3-fold (150 μm to 440 μm).

But when there were many gj-channels (300, 1000, or 3000), there was substantial ΔV_m _in the adjoining cells and beyond, as can be Figure 6. This voltage decay was exponential, as demonstrated in Figure 7, in which the ΔV_m _is plotted on a logarithmic scale. A straight line on such a semilog plot indicates that the voltage fall-off is exponential, with the slope of the straight line being indicative of *λ*. *λ *is the distance at which the ΔV_m _falls to 1/e or 36.8% of the initial value (at x = 0). As indicated in Figure 7, the *λ *values were 150 μm, 270 μm, and 440 μm with 300, 1000, and 3000 gj-channels, respectively (see Table 5). Thus, the *λ *value increases nearly in proportion to the square root of the number of gj-channels.

**Table 5:** Summary of data showing the effect of the number of gj-channels on the length constant (*λ*) and on overall propagation velocity (*θ*_ov_) in the single linear chain of 100 myocardial cells

	**No. of gj-channels**	N MathType@MTEF@5@5@+=feaafiart1ev1aaatCvAUfKttLearuWrP9MDH5MBPbIqV92AaeXatLxBI9gBaebbnrfifHhDYfgasaacH8akY=wiFfYdH8Gipec8Eeeu0xXdbba9frFj0=OqFfea0dXdd9vqai=hGuQ8kuc9pgc9s8qqaq=dirpe0xb9q8qiLsFr0=vr0=vr0dc8meaabaqaciaacaGaaeqabaqabeGadaaakeaadaGcaaqaaiabd6eaobWcbeaaaaa@2DEC@	***λ *(μm)**	*θ*_ov _**(cm/sec)**
A	300	17.3	150	358
B	1000	31.6	270	874
C	3000	54.8	440	1850
	**Ratios**			
C/A	10.0	3.17	2.93	5.17
C/B	3.00	1.73	1.63	2.12
B/A	3.33	1.82	1.80	2.44

N = Number of gj-channels.

The data are given for only those 3 cases in which there was an exponential decay of potential as a function of distance.

Note that the ratios for the effects were always substantially higher for *θ*_ov _than for *λ*. That is, adding gj-channels had a larger effect on *θ*_ov _than on *λ*.

Also note that *λ *varies approximately with the square root of the number of gj-channels.

#### 2. Input resistance (R_in_) measurement

**Figure 8:**
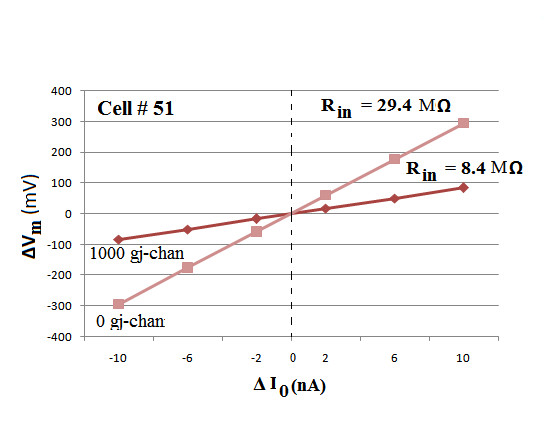
Voltage/current curves obtained for myocardial cell # 51 near the middle of the single linear 100-cell chain. Depolarizing and hyperpolarizing rectangular current pulses (duration of 5 ms and intensities of 2, 6, and 10 nA) were injected intracellularly into cell #51 and the resultant voltage changes in that cell were recorded and plotted. The ΔV_0_/I_0 _curves were linear, in both the depolarizing and hyperpolarizing sectors, because rectification was not incorporated into the basic membrane units that composed each cell. The number of gj-channels connecting the contiguous cells was varied from zero to 3000, but only two of the curves are illustrated, namely for zero and 1,000 gj-channels. As predicted, the curve for 0 gj-channels had a steeper slope and higher input resistance (R_in_) than the curve for 1000 gj-channels, namely 29.4 MΩ versus 8.4 MΩ.

To measure the input resistance (R_in_) of the chain of cells, different intensities of current (rectangular pulses) were applied intracellularly into cell #51 of the 100-cell chain, and the resulting ΔV_m _was measured. All of these data are given in Table 6. The data for 1000 gj-channels are plotted in Figure 8. The curve is exactly linear, as expected because rectifying properties were not incorporated into the basic circuit units. In addition, hyperpolarizing and depolarizing currents gave comparable results. The slope of the line plotted gives the input resistance (R_in _= ΔV_o_/I_o_, where ΔV_o _is the voltage change at distance x = 0). The measured value for this case (1000 gj-channels) is 8.4 MΩ. The R_in _value at zero gj-channels is 29.4 MΩ, a value close to that measured physiologically [21,22]. Table 6 gives the R_in _values obtained for all numbers of gj-channels. These values can also be discerned from the ΔV_m _values measured in cell #51 presented in Figure 6.

**Table 6:** Input resistance (R_in_) measurements in the injected cell (cell #51) of the 100-cell chain

No. of gj- Channels	ΔV_m _in cell #51 (mV)	R_in _(MΩ)
0	294	29.4
10	278	27.8
30	253	25.3
100	200	20
300	138	13.8
1000	84	8.4
3000	51	5.1

I_o _injected was 10.0 nA

Rin=ΔVoIo
 MathType@MTEF@5@5@+=feaafiart1ev1aaatCvAUfKttLearuWrP9MDH5MBPbIqV92AaeXatLxBI9gBaebbnrfifHhDYfgasaacH8akY=wiFfYdH8Gipec8Eeeu0xXdbba9frFj0=OqFfea0dXdd9vqai=hGuQ8kuc9pgc9s8qqaq=dirpe0xb9q8qiLsFr0=vr0=vr0dc8meaabaqaciaacaGaaeqabaqabeGadaaakeaacqWGsbGudaWgaaWcbaGaemyAaKMaemOBa4gabeaakiabg2da9maalaaabaGaeuiLdqKaemOvay1aaSbaaSqaaiabd+gaVbqabaaakeaacqWGjbqsdaWgaaWcbaGaem4Ba8gabeaaaaaaaa@38CB@

The resistance value measured in the adjacent cells is known as the polarization resistance (R_p_), and is proportional to the ΔV_m _measured in those cells. For example, the R_p _values shown in Figure 7 (1000 gj-channels) are 4.7 MΩ for cell #52 (and 50), and 2.6 MΩ for cell #53 (and 49).

The equation for calculating R_in _is:

Rin=ΔV0I0
 MathType@MTEF@5@5@+=feaafiart1ev1aaatCvAUfKttLearuWrP9MDH5MBPbIqV92AaeXatLxBI9gBaebbnrfifHhDYfgasaacH8akY=wiFfYdH8Gipec8Eeeu0xXdbba9frFj0=OqFfea0dXdd9vqai=hGuQ8kuc9pgc9s8qqaq=dirpe0xb9q8qiLsFr0=vr0=vr0dc8meaabaqaciaacaGaaeqabaqabeGadaaakeaacqWGsbGudaWgaaWcbaGaemyAaKMaemOBa4gabeaakiabg2da9maalaaabaGaeuiLdqKaemOvay1aaSbaaSqaaiabicdaWaqabaaakeaacqWGjbqsdaWgaaWcbaGaeGimaadabeaaaaaaaa@37D9@

**Ω **= **Ω**.

where ΔV_0 _is the voltage change at the site of current injection (x = 0) and I_0 _is the amount of current injected (at x = 0). The equation relating R_in _and *λ *in a cable is:

Rin=12(ri+rj)λ
 MathType@MTEF@5@5@+=feaafiart1ev1aaatCvAUfKttLearuWrP9MDH5MBPbIqV92AaeXatLxBI9gBamXvP5wqSXMqHnxAJn0BKvguHDwzZbqegyvzYrwyUfgarqqtubsr4rNCHbGeaGqiA8vkIkVAFgIELiFeLkFeLk=iY=Hhbbf9v8qqaqFr0xc9pk0xbba9q8WqFfeaY=biLkVcLq=JHqVepeea0=as0db9vqpepesP0xe9Fve9Fve9GapdbaqaaeGacaGaaiaabeqaamqadiabaaGcbaGaemOuai1aaSbaaSqaaiabdMgaPjabd6gaUbqabaGccqGH9aqpdaWcaaqaaiabigdaXaqaaiabikdaYaaacqGGOaakcqWGYbGCdaWgaaWcbaGaemyAaKgabeaakiabgUcaRiabdkhaYnaaBaaaleaacqWGQbGAaeqaaOGaeiykaKccciGae83UdWgaaa@4E53@

Ω=(Ωcm+Ωcm)cm=Ω
 MathType@MTEF@5@5@+=feaafiart1ev1aaatCvAUfKttLearuWrP9MDH5MBPbIqV92AaeXatLxBI9gBaebbnrfifHhDYfgasaacH8akY=wiFfYdH8Gipec8Eeeu0xXdbba9frFj0=OqFfea0dXdd9vqai=hGuQ8kuc9pgc9s8qqaq=dirpe0xb9q8qiLsFr0=vr0=vr0dc8meaabaqaciaacaGaaeqabaqabeGadaaakeaacqqHPoWvcqGH9aqpcqGGOaakdaWcaaqaaiabfM6axbqaaiabdogaJjabd2gaTbaacqGHRaWkdaWcaaqaaiabfM6axbqaaiabdogaJjabd2gaTbaacqGGPaqkcqWGJbWycqWGTbqBcqGH9aqpcqqHPoWvaaa@3FBA@

where r_i _is the internal longitudinal resistance in Ω/cm, and r_j _is the junctional resistance in Ω/cm. The 1/2 factor reflects the fact that the injected current spreads in both directions from the point of injection. Since

λ=rmri+r0
 MathType@MTEF@5@5@+=feaafiart1ev1aaatCvAUfKttLearuWrP9MDH5MBPbIqV92AaeXatLxBI9gBaebbnrfifHhDYfgasaacH8akY=wiFfYdH8Gipec8Eeeu0xXdbba9frFj0=OqFfea0dXdd9vqai=hGuQ8kuc9pgc9s8qqaq=dirpe0xb9q8qiLsFr0=vr0=vr0dc8meaabaqaciaacaGaaeqabaqabeGadaaakeaaiiGacqWF7oaBcqGH9aqpdaGcaaqaamaalaaabaGaemOCai3aaSbaaSqaaiabd2gaTbqabaaakeaacqWGYbGCdaWgaaWcbaGaemyAaKgabeaakiabgUcaRiabdkhaYnaaBaaaleaacqaIWaamaeqaaaaaaeqaaaaa@38FA@

cm=Ω.cmΩcm+Ωcm=cm2
 MathType@MTEF@5@5@+=feaafiart1ev1aaatCvAUfKttLearuWrP9MDH5MBPbIqV92AaeXatLxBI9gBaebbnrfifHhDYfgasaacH8akY=wiFfYdH8Gipec8Eeeu0xXdbba9frFj0=OqFfea0dXdd9vqai=hGuQ8kuc9pgc9s8qqaq=dirpe0xb9q8qiLsFr0=vr0=vr0dc8meaabaqaciaacaGaaeqabaqabeGadaaakeaacqWGJbWycqWGTbqBcqGH9aqpdaGcaaqaamaalaaabaGaeuyQdCLaeiOla4Iaem4yamMaemyBa0gabaWaaSaaaeaacqqHPoWvaeaacqWGJbWycqWGTbqBaaGaey4kaSYaaSaaaeaacqqHPoWvaeaacqWGJbWycqWGTbqBaaaaaaWcbeaakiabg2da9maakaaabaGaem4yamMaemyBa02aaWbaaSqabeaacqaIYaGmaaaabeaaaaa@4426@

where r_m _is the membrane resistance in Ω-cm. Then assuming r_0_, the external longitudinal resistance, to be zero:

Rin=12[ri+rjri]rm
 MathType@MTEF@5@5@+=feaafiart1ev1aaatCvAUfKttLearuWrP9MDH5MBPbIqV92AaeXatLxBI9gBaebbnrfifHhDYfgasaacH8akY=wiFfYdH8Gipec8Eeeu0xXdbba9frFj0=OqFfea0dXdd9vqai=hGuQ8kuc9pgc9s8qqaq=dirpe0xb9q8qiLsFr0=vr0=vr0dc8meaabaqaciaacaGaaeqabaqabeGadaaakeaacqWGsbGudaWgaaWcbaGaemyAaKMaemOBa4gabeaakiabg2da9maalaaabaGaeGymaedabaGaeGOmaidaamaadmaabaWaaOaaaeaacqWGYbGCdaWgaaWcbaGaemyAaKgabeaaaeqaaOGaey4kaSYaaSaaaeaacqWGYbGCdaWgaaWcbaGaemOAaOgabeaaaOqaamaakaaabaGaemOCai3aaSbaaSqaaiabdMgaPbqabaaabeaaaaaakiaawUfacaGLDbaadaGcaaqaaiabdkhaYnaaBaaaleaacqWGTbqBaeqaaaqabaaaaa@42D3@

Ω=[Ωcm+ΩcmΩcm]Ω.cm=[Ωcm+Ωcm]Ω.cm=Ω+Ω=Ω
 MathType@MTEF@5@5@+=feaafiart1ev1aaatCvAUfKttLearuWrP9MDH5MBPbIqV92AaeXatLxBI9gBaebbnrfifHhDYfgasaacH8akY=wiFfYdH8Gipec8Eeeu0xXdbba9frFj0=OqFfea0dXdd9vqai=hGuQ8kuc9pgc9s8qqaq=dirpe0xb9q8qiLsFr0=vr0=vr0dc8meaabaqaciaacaGaaeqabaqabeGadaaakeaacqqHPoWvcqGH9aqpdaWadaqaamaakaaabaWaaSaaaeaacqqHPoWvaeaacqWGJbWycqWGTbqBaaaaleqaaOGaey4kaSYaaSaaaeaadaWcaaqaaiabfM6axbqaaiabdogaJjabd2gaTbaaaeaadaGcaaqaamaalaaabaGaeuyQdCfabaGaem4yamMaemyBa0gaaaWcbeaaaaaakiaawUfacaGLDbaadaGcaaqaaiabfM6axjabc6caUiabdogaJjabd2gaTbWcbeaakiabg2da9maadmaabaWaaOaaaeaadaWcaaqaaiabfM6axbqaaiabdogaJjabd2gaTbaaaSqabaGccqGHRaWkdaGcaaqaamaalaaabaGaeuyQdCfabaGaem4yamMaemyBa0gaaaWcbeaaaOGaay5waiaaw2faamaakaaabaGaeuyQdCLaeiOla4Iaem4yamMaemyBa0galeqaaOGaeyypa0JaeuyQdCLaey4kaSIaeuyQdCLaeyypa0JaeuyQdCfaaa@5E4C@

Since in the experiments on length constant, the membrane resistance (r_m_) and the internal longitudinal resistance (r_i_) were not altered, whereas r_j _was lowered by insertion of gj-channels, then R_in _should be reduced, as shown in Figures 6, 7 and 8.

### C. Propagation velocity vs. length constant (100-cell chain)

**Figure 9:**
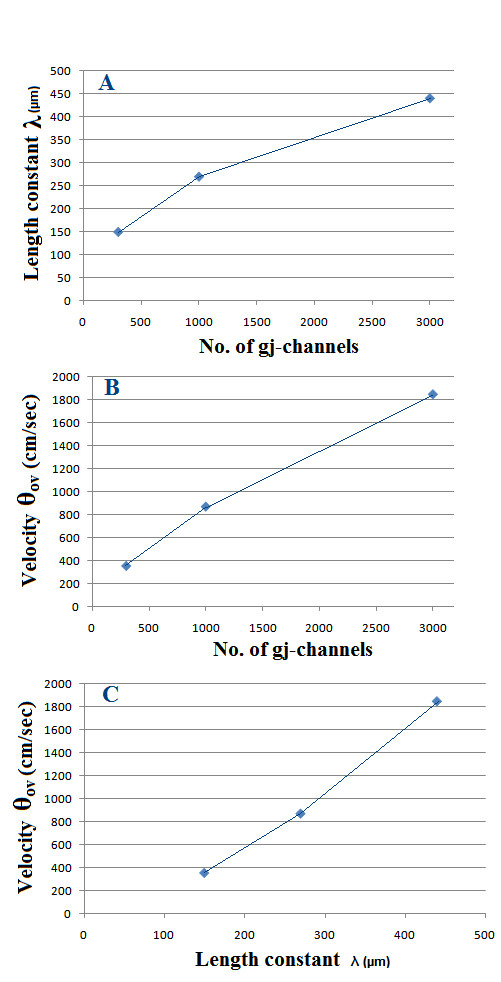
Graphic plots for the case where there were many gj-channels (namely 300, 1000, and 3000), giving an exponential fall-off in voltage. **A: **Length constant (*λ*) as a function of the number of gj-channels. *λ *varies approximately with the square root of the number of gj-channels. **B: **Overall propagation velocity (*θ*_ov_) as a function of the number of channels. **C: **Velocity (*θ*_ov_) plotted against *λ*, showing that approximate doubling or tripling of *λ *produces a greater effect of propagation velocity.

The relationship between *λ *and number of gj-channels is depicted in Figure 9A. This should be compared with Figure 3, which shows the relationship between propagation velocity and number of gj-channels. This same relationship is shown in Figure 9B, but only for those three cases (300, 1000, and 3000 gj-channels) in which there was an exponential fall-off in ΔV_m_. As shown, propagation velocity varies nearly linear with the number of gj-channels. However, as stated previously, *λ *varies approximately with the square root of the number of gj-channels (Table 5). Compare the ratios in the √N column with those in the *λ *column in Table 5. Thus, increasing the number of gj-channels has a great effect on propagation velocity, whereas it has a smaller effect on length constant. Figure 9C gives a plot of the relationship between propagation velocity and length constant.
